# Isolation and Molecular Characterization of Biofouling Bacteria and Profiling of Quorum Sensing Signal Molecules from Membrane Bioreactor Activated Sludge

**DOI:** 10.3390/ijms15022255

**Published:** 2014-02-04

**Authors:** Harshad Lade, Diby Paul, Ji Hyang Kweon

**Affiliations:** Department of Environmental Engineering, Konkuk University, Seoul-143-701, Korea; E-Mail: harshadlade@gmail.com

**Keywords:** membrane bioreactor, activated sludge, isolation of bacteria, violacein assay, β-galactosidase assay, quorum sensing signals, biofilm formation

## Abstract

The formation of biofilm in a membrane bioreactor depends on the production of various signaling molecules like *N*-acyl homoserine lactones (AHLs). In the present study, a total of 200 bacterial strains were isolated from membrane bioreactor activated sludge and screened for AHLs production using two biosensor systems, *Chromobacterium violaceum* CV026 and *Agrobacterium tumefaciens* A136. A correlation between AHLs production and biofilm formation has been made among screened AHLs producing strains. The 16S rRNA gene sequence analysis revealed the dominance of *Aeromonas* and *Enterobacter sp*. in AHLs production; however few a species of *Serratia*, *Leclercia*, *Pseudomonas*, *Klebsiella*, *Raoultella* and *Citrobacte*r were also identified. The chromatographic characterization of sludge extract showed the presence of a broad range of quorum sensing signal molecules. Further identification of sludge AHLs by thin layer chromatography bioassay and high performance liquid chromatography confirms the presence of C4-HSL, C6-HSL, C8-HSL, 3-oxo-C8-HSL, C10-HSL, C12-HSL, 3-oxo-C12-HSL and C14-HSL. The occurrence of AHLs in sludge extract and dominance of *Aeromonas* and *Enterobacter sp.* in activated sludge suggests the key role of these bacterial strains in AHLs production and thereby membrane fouling.

## 1. Introduction

Membrane bioreactors (MBR) have become state-of-the-art in wastewater treatment and have been in commercial use for more than two decades. The membrane filtration unit present in the MBR retains biomass and makes it effective for widespread use [1]. However, similar to other membrane separation technologies, membrane fouling still remains a major obstruction that limits its widespread use [2]. The occurrence of biofilm in MBR leads to membrane clogging and results in low treatment efficiency. Controlling biofilm formation in membranes can prevent MBR failures, reduce environmental damage and minimize revenue loss. In order to overcome the fouling problem, several operational strategies have been employed; however membrane fouling remains an issue of investigation in MBR operation.

Membrane biofouling is caused by several physicochemical and biological processes, which are highly dependent on composition of feedwater, membrane characteristics, operation conditions and microorganisms present [3]. The biofilm can be formed by a single bacterial species, but MBR biofilms almost always consist of mixed cultured communities from different genera. Therefore, it is essential to know the microbial community structure as a prerequisite for fundamental understanding of the biofouling phenomenon in MBR [4]. In addition, characterization of individual microbial genera will also give important information about bacteria involved in biofouling and therefore aid the design of control strategies. Culture-dependent techniques have been previously used for the isolation of a wide range of heterotrophic bacteria from activated sludge. Lim *et al*. [4] isolated a total of 61 bacterial species from membrane bioreactor biocake using culture-dependent techniques with various growth mediums. Specifically, they reported the predominance of *Enterobacter* and *Dyella* genera in MBR biocake bacterial isolates with a high number of *E. cancerogenus* strains. Biofouling bacteria typically present in activated sludge include the *Alpha-*, *Beta-* and *Gammaproteobacteria*, as well as the *Bacteroidetes* and the *Actinobacteria* [5]. The occurrence of different bacterial species from the genera *Aeromonas*, *Acinetobacter*, *Citrobacter*, *Klebsiella*, *Neisseria*, *Malikia* and *Pseudomonas lineages* have also been reported in activated sludge [6]. Miura *et al*. [3] found that *Betaproteobacteria* probably played the major role in development of mature biofilms in MBR treating municipal wastewater.

Biofilm formation begins with the colonization of bacteria and subsequent surface attachment. The initial colonization starts with the detection of quorum sensing (QS) signal molecules from the surrounding environment. The QS regulates the bacterial group behaviors in a cell density dependent way and is closely associated with biofouling of the membrane in MBR treating wastewater [7]. Thus characterization of activated sludge AHLs will provide an additional insight into the bacteria involved in MBR. Recently, Yeon *et al*. [7] reported three different AHLs: C6-HSL, C8-HSL and 3-oxo-C8-HSL in mixed cultured biocake of MBR treating wastewater. In addition, existence of C4-HSL and C6-HSL has been reported in soil isolate *Burkholderia sp.* strain A9 [8].

Developing more effective strategies to deal with the biofouling problem in MBRs treating wastewater still requires fundamental investigations on all aspects of biofilm formation. Several attempts have been made to know the physicochemical and biological parameters that lead to biofilm formation. Compared with physicochemical parameters, investigating the bacterial diversity responsible for MBR biofouling has been the main focus for future control strategies since these are the basic cause of membrane fouling. However, the microbial community responsible for biofouling is still not well characterized. To understand the bacteria responsible for biofouling and to choose the most appropriate control strategies, the isolation and molecular characterization of MBR activated sludge bacteria is essential. In addition, thorough exploration of the phylogenetic relationships within sludge bacteria and characterization of QS signal molecules will also provide additional insight into the actual fouling problem. However, such investigations do not allow for a reliable measure of microbial abundance and diversity of species, because the majority of the microbial population from the environment cannot be cultured [9]. But the approximate estimation and characterization of AHLs producing and biofouling bacterial diversity is essential for designing effective quorum sensing inhibitors (QSI) based control technologies.

The primary objectives of the present study were to characterize the key bacterial community responsible for membrane biofouling in a MBR treating wastewater, and profiling of QS signal molecules involved. In view of this, the culture-dependent isolation of activated sludge bacteria were carried out and initial screening for AHLs producing bacterial strains was performed with two wide range AHLs biosensor systems *C. violaceum* CV026 and *A. tumefaciens* A136. The correlation between AHLs production and biofilm formation among screened AHLs producing bacterial isolates has been made. The bacteria responsible for AHLs production and subsequent biofilm formation were identified by 16S rRNA gene sequence analysis and a phylogenetic relationship was established. The characterization of sludge AHLs was also performed by TLC and HPLC analysis, which will help us to design and investigate the QSI strategies for control of membrane biofouling.

## 2. Results and Discussion

### 2.1. Isolation and Screening of AHLs Producing Bacteria

**Figure 1.**
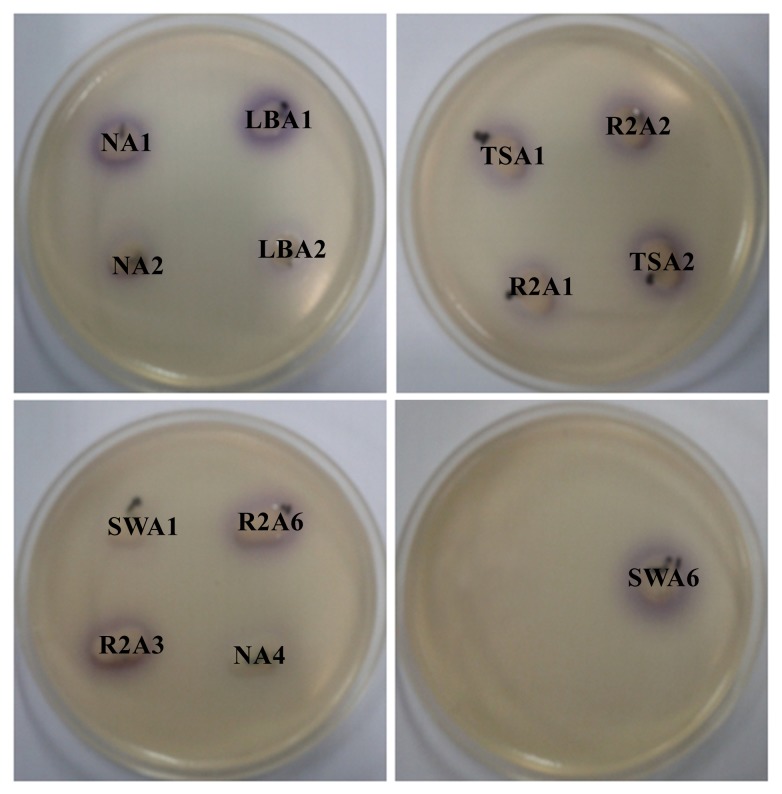
Screening of bacterial isolates for AHLs production using agar-plate well diffusion assay with *C. violaceum* CV026. All the thirteen isolates producing short and medium-chain AHLs are shown.

**Figure 2.**
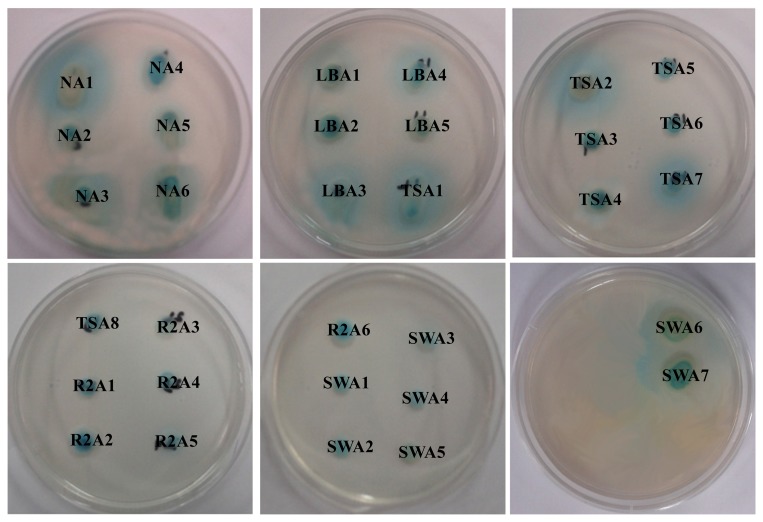
Screening of bacterial isolates for AHLs production using agar-plate well diffusion assay with *A. tumefaciens* A136. All the thirty two isolates producing medium and long-chain AHLs are shown.

Isolation and characterization of AHLs producing bacteria becomes essential in the development of membrane biofouling control strategies. The culture dependent techniques allowed the isolation of a wide range of heterotrophic bacteria from environmental samples like activated sludge and marine sponges [4,10]. In the present study, a total of 200 morphologically distinct bacterial strains were randomly isolated from MBR activated sludge using culture-dependent techniques with NB, LB, TSB, R2A and SW medium respectively. Lim *et al*. [4] has isolated the QS active microbes from lab scale MBR using culture dependent techniques and correlated the microbial community with biofouling in MBR. Two different biosensor systems specific for short-chain AHLs (*C. violaceum* CV026) and long-chain AHLs (*A. tumefaciens* A136) were employed for screening AHLs producing bacterial strains. As a result, a total of 32 bacterial isolates were found to produce AHLs based on the development of purple and blue coloration in *C. violaceum* CV026 and *A. tumefaciens* A136 reporter strains, respectively. The well diffusion bioassay results of AHLs producing bacterial isolates are summarized in Table 1. The presence of 32 AHLs-producing and 168 non AHLs-producing strains in activated sludge sample isolates represent a model high-density microbial community of biotechnological importance. Among the 32 AHLs producers, 13 bacterial strains showed the ability to produce short or medium-chain AHLs with the induction of violacein production in reporter strain *C. violaceum* CV026 (Figure 1). However, all the 32 bacterial strains produce medium or long-chain AHLs as indicated by β-galactosidase activity with *A. tumefaciens* A136 (Figure 2). Similarly, Chong *et al*. [6] isolated and screened 52 morphologically distinct strains from activated sludge for production of QS signal molecules using various bioassays.

### 2.2. Biofilm Formation among AHLs Producing Bacterial Isolates

**Figure 3.**
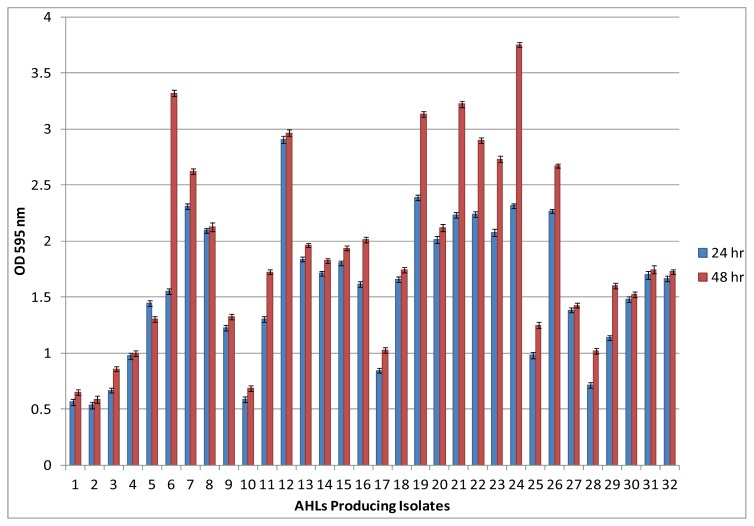
Biofilm formation among the AHLs producing bacteria as accessed by microtiter plate assay. The values are expressed as mean of four experiments and error bars indicates the standard deviation.

Most bacteria are social microorganisms that live in terrestrial environments and form highly organized and complex communities in the form of biofilms. Such biofilms develop on various surfaces including membrane bioreactor treating wastewater. It has been observed that all the 32 AHLs producing bacterial isolates form biofilms on polystyrene surfaces within 24 h incubation as assessed by the 96-well microplate assay. Quantification of biofilm produced by the 32 AHLs producing bacterial isolates revealed that all the strains seem to have different biofilm-forming abilities. Further prolonged incubation up to 48 h showed significant increases in biofilm formation by isolate no 6, 19, 21, 22, 23, 24 and 26 as compare to 24 h incubation (Figure 3). The ability of bacteria to establish biofilm can be used as a strategy for survival or for colonization on membrane surfaces [11,12]. Several recent studies have addressed that quorum sensing is the key factor which regulates biofilm formation by means of motility, adhesion, maturation and dispersal [13–16]. The present biofilm formation results are in good agreement with the previous studies which can establish the correlation between QS signal production and biofilm formation.

### 2.3. Identification of AHLs Producing Bacteria

The accurate identification of bacterial isolates up to species level is crucial since this gives an insight into the bacterial diversity of the activated sludge. The identification of AHLs producing, as well as biofilm forming, bacterial isolates in this study was carried out by advanced molecular biology technique based on 16S rRNA gene sequence analysis. Analysis of nucleotide sequences has proved to be a powerful technique for phylogenetic characterization of bacteria [17]. The nucleotide blast analysis of complete 16S rRNA gene sequences form 32 bacterial isolates with the sequences available in the NCBI database demonstrated that they belong to eight different genera. The bacterial sequences comparison showed 98%–100% identification similarities with 16S rRNA gene sequences of the closest relative strains at NCBI database. The present molecular characterization reveals the significant differences in genetic diversity among AHLs producing bacterial strains. BLAST results of the 16S rRNA gene sequence analysis suggest that twelve isolates belong to genus *Aeromonas* (NA1, NA2, NA3, NA5, NA6, LBA1, LBA2, TSA1, TSA2, R2A1, R2A2, R2A3), ten to *Enterobacter* (NA4, LBA3, LBA4, LBA5, TSA5, R2A4, R2A6, SWA1, SWA3, SWA5), three each to *Serratia* (STSA8, SWA6, SWA7) and *Leclercia* (TSA4, TSA6, SWA4) while only one isolate was assigned each to *Pseudomonas* (TSA3), *Klebsiella* (SWA2), *Raoultella* (TSA7) and *Citrobacter* (R2A5) (Table 2). Molecular characterization study suggest the dominance of culturable *Aeromonas* and *Enterobacter* species, which are known to be common AHLs producing bacteria present in the membrane bioreactor activated sludge. Although most of the bacterial isolates belong to two dominant genera *Aeromonas* and *Enterobacter*, the molecular identification revealed some significant differences at the species level. Our results are in good agreement with those of Chong *et al*. who demonstrated distinct strains isolated from activated sludge have various genera and produce broad range of AHLs, which includes 17 *Aeromonas*, six *Acinetobacter*, five *Citrobacter*, four *Klebsiella*, two *Neisseria*, two *Malikia* and two *Pseudomonas* [6].

### 2.4. Phylogenetic Analysis

**Figure 4.**
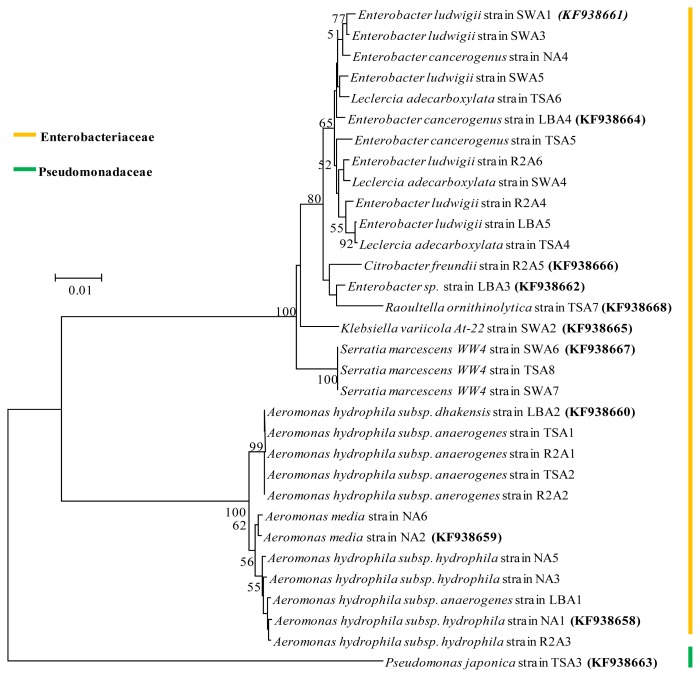
Phylogenetic relationship of the AHLs producing bacteria isolated from membrane bioreactor activated sludge. The tree was constructed using neighbor joining algorithm with Kimura 2 parameter distances in MEGA 4.0 software. Numbers at the nodes indicate percent bootstrap values above 50 supported by 550 replicates. The bar indicates the Juke-Cantor evolutionary distance.

Based on assembled complete 16S rRNA gene sequence alignment, the phylogeny tree of 32 culturable AHLs producing bacterial isolates was constructed with the neighbor-joining method. This method demonstrates the position of each bacterial isolate in the phylogeny with bootstrap support. The result from the well-supported phylogeny with high resolution inner branches suggests the existence of 31 bacterial strains from *Enterobacteriaceae* family and only one from *Pseudomonadaceae* (Figure 4). Phylogenetic analysis also reveals that the isolate *Pseudomonas japonica* strain TSA3 forms a distinct clade and possibly belongs to a novel species with the *Pseudomonas japonica* strain IAM15071 as its closest relative. The *Aeromonas* and *Enterobacter* were dominant groups, which constituted 37% and 31% of the total AHLs producing isolates respectively. *Serratia* and *Leclercia* were the next group and constituted 9%, whereas *Pseudomonas*, *Klebsiella*, *Raoultella* and *Citrobacter* constituted 3% each of total AHLs producing strains. Similar dominance by these groups was also reported in some AHLs producing bacterial isolation studies, where *Aeromonas* and *Enterobacter* species were found as dominant genera in bacterial quorum sensing signal producers [4,6,18]. Bacteria from the genus *Aeromonas*, *Pseudomonas*, *Enterobacter*, *Klebsiella*, *Serratia* and *Citrobacter* are well known in AHLs production and biofilm formation.

### 2.5. Nucleotide Sequence Accession Numbers

The eleven distinguishable AHLs producing bacterial isolates complete 16S rRNA gene sequences have been deposited in the GenBank database under the accession numbers: KF938658 (*Aeromonas hydrophila subsp. hydrophila* strain NA1), KF938659 (*Aeromonas media* strain NA2), KF938660 (*Aeromonas hydrophila subsp. dhakensis* strain LBA2), KF938661 (*Enterobacter ludwigi* strain SWA1), KF938662 (*Enterobacter sp.* strain LBA3), KF938663 (*Pseudomonas japonica* strain TSA3), KF938664 (*Enterobacter cancerogenus* strain LBA4), KF938665 (*Klebsiella variicola* strain SWA2), KF938666 (*Citrobacter freundii* strain R2A5), KF938667 (*Serratia marcescens* strain SWA6) and KF938668 (*Raoultella ornithinolytica* strain TSA7).

### 2.6. Extraction and Characterization of Sludge AHLs

**Figure 5.**
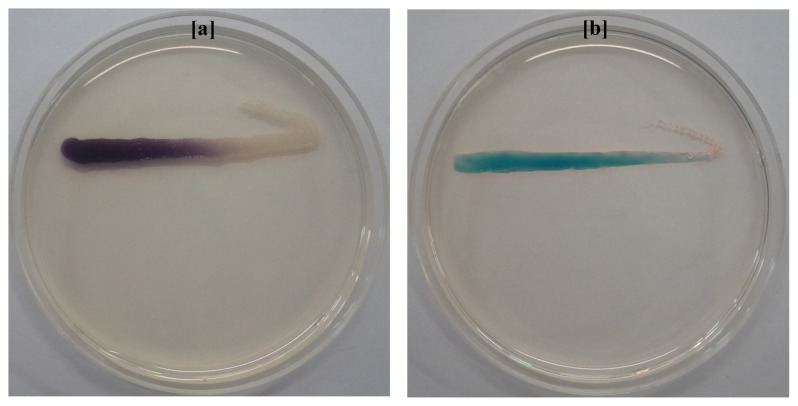
Detection of AHLs extracted from activated sludge by agar-plate cross feeding violacein and β-galactosidase assay with *C. violaceum* CV026 and *A. tumefaciens* A136 respectively. (**a**) Evidence for the presence of short and medium-chain AHLs in activated sludge extract is indicated by purple coloration of the reporter strain *C. violaceum* CV026; (**b**) Evidence for the presence of medium and long-chain AHLs activated sludge extract is indicated by blue coloration of the reporter strain *A. tumefaciens* A136.

Characterization of sludge AHLs is becoming important since future biofouling control strategies will be designed by considering QS inhibition. In the present study, AHLs extracted from MBR activated sludge with acidified ethyl acetate were initially analyzed for the presence of a broad spectrum of AHLs with two biosensor strains. The cross-feeding agar plate assay with *C. violaceum* CV026 and *A. tumefaciens* A136 confirms the presence of a broad-range of AHLs in activated sludge extract (Figure 5). This method has the advantage of rapid detection of AHLs with approximate types.

**Figure 6.**
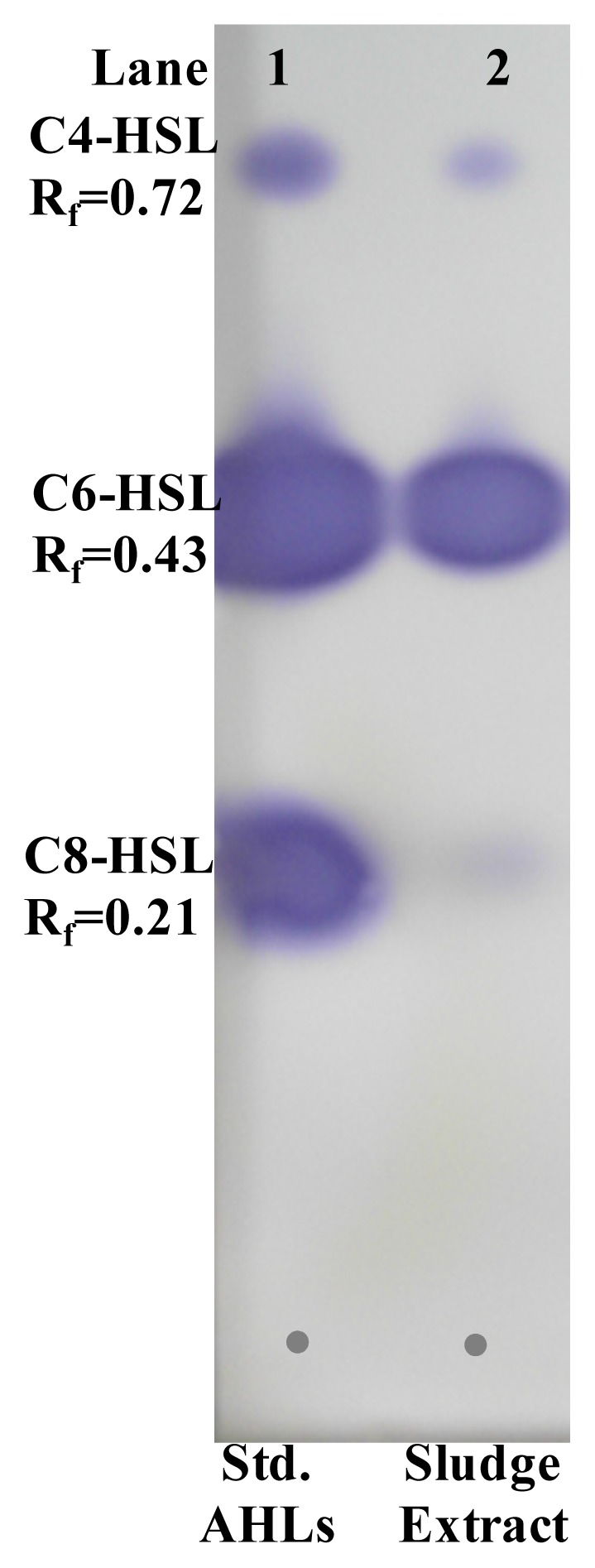
TLC bioassay with *C. violaceum* CV026 for the identification of AHLs extracted from activated sludge. Lane 1: Standard AHLs (0.5 μg/μL); C4-HSL, C6-HSL and C8-HSL. Lane 2: Extract of the activated sludge (20 μL). Tentative identification of the sludge extracted AHLs based on migration of standards, is indicated.

Separation of AHLs by TLC bioassay with reporter strains gives an identifying index of AHLs present in the sludge extract. The TLC chromatogram overlayed with biosensor strains *C. violaceum* CV026 showed three well differentiated spots indicating the presence of short-chain AHLs in activated sludge extracts. Relative retention factor (Rf) of developed purple colour spots were calculated and compared with those of standard AHLs. The crude sludge AHLs extracts showed close resemblance with standard C4-HSL (Rf = 0.72), C6-HSL (Rf = 0.43) and C8-HSL (Rf = 0.21) (Figure 6). Further TLC analysis for long-chain AHLs by β-galactosidase assay with *A. tumefaciens* A136 showed a tailing pattern for sludge extracted AHLs and there was no clear separation of spots (data not shown).

**Figure 7.**
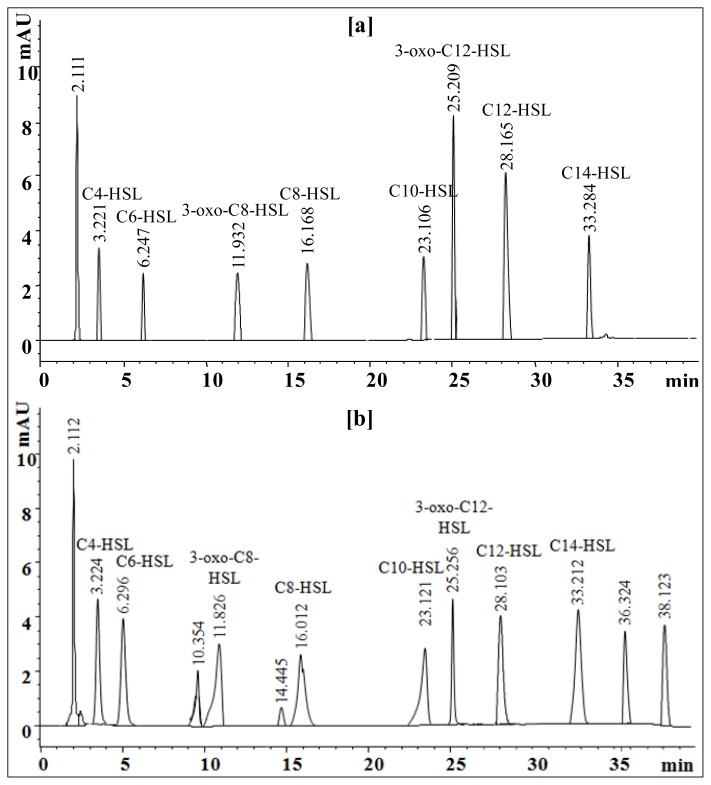
Identification of the AHLs extracted from MBR activated sludge by HPLC. (**a**) Chromatogram showing the retention times of standard AHLs each at 50 μg/mL concentration in a mixture of C4-HSL, C6-HSL, C8-HSL, 3-oxo-C8-HSL, C10-HSL, C12-HSL, 3-oxo-C12-HSL and C14-HSL; (**b**) The sludge AHLs were identified by comparing appeared peaks retention time with those of respective standard AHLs.

HPLC analysis was performed to identify sludge AHLs by comparing appeared peaks retention time with those of respective standard AHLs. The standard AHLs showed retention times of 3.221 for C4-HSL, 6.247 for C6-HSL, 11.932 for 3-oxo-C8-HSL, 16.168 for C8-HSL, 23.106 for C10-HSL, 25.209 for 3-oxo-C12-HSL, 28.165 for C12-HSL and 33.284 for C14-HSL (Figure 7a). The HPLC chromatogram of sludge extract showed a total of 13 peaks at various retention times. Eight peaks appeared with the retention times of 3.224 for C4-HSL, 6.296 for C6-HSL, 11.826 for 3-oxo-C8-HSL, 16.012 for C8-HSL, 23.121 for C10-HSL, 25.256 for 3-oxo-C12-HSL, 28.103 for C12-HSL and 33.212 for C14-HSL are identical to standard AHLs (Figure 7b). Furthermore, appearance of minor peaks at retention times of 10.354 and 14.445 min and the major peaks at 36.324 and 38.123 min that do not resemble standard AHLs suggest the presence of unidentified AHLs or microbial metabolites in the sludge extract. The appearance of the first major peak at retention time of 2.112 was for solvent. From the HPLC analyses it can be concluded that the presence of eight broad spectrum AHLs in the activated sludge sample may be due to the active involvement of AHLs producing bacteria from the family *Enterobacteriaceae* and *Pseudomonadaceae* as confirmed by 16S rRNA gene sequence analysis and phylogenetic relationship.

## 3. Experimental Section

### 3.1. Chemicals and Microbiological Media

HPLC grade ethyl acetate, methanol, acetonitrile, water and dimethylformamide were purchased from Fisher Scientific (Fairlawn, NJ, USA). 5-bromo-4-chloro-3-indolyl-β-d-galactopyranoside (X-gal), kanamycin sulfate, spectinomycin and tetracycline were obtained from Sigma-Aldrich (St. Louis, MO, USA). Dehydrated culture media used includes Luria-Bertani (LB), Nutrient Broth (NB), Tryptic Soy Broth (TSB) and Reasoner’s 2A (R2A) broth were procured from BD-Difco (Franklin Lakes, NJ, USA). All the chemicals used were of highest analytical grade.

### 3.2. Standard N-acyl Homoserine Lactones

Standard AHLs viz. *N*-butanoyl-l-homoserine lactone (C4-HSL), *N*-hexanoyl-l-homoserine lactone (C6-HSL), *N*-octanoyl-l-homoserine lactone (C8-HSL), 3-oxo-octanoyl-l-homoserine lactone (3-oxo-C8-HSL) and *N*-decanoyl-l-homoserine (C10-HSL) were purchased from Sigma-Aldrich (St. Louis, MO, USA). The other long-chain AHLs *N*-dodecanoyl-l-homoserine (C12-HSL), *N*-(3-oxo-dodecanoyl)-l-homoserine lactone (3-oxo-C12-HSL) and *N*-tetradecanoyl-l-homoserine lactone (C14-HSL) were purchased from the Cayman Chemical Company (Ann Arbor, MI, USA).

### 3.3. Quorum Sensing Reporter’s Strains

Two different AHL biosensor systems *C. violaceum* CV026 (Mini-Tn5 mutant of ATCC31532) and *A. tumefaciens* A136 which are deficient in AHLs production were used to screen the wide range of AHLs producing bacterial isolates. These biosensor strains detect and respond to a range of exogenous AHLs molecules with an acyl side chain of four to fourteen carbons by inducing the synthesis of the purple pigment violacein and blue coloration by β-galactosidase activity. *C. violaceum* 026 bears a LuXR homologue, CviR, which regulates the production of violacein, a purple pigment when induced by C4-HSL, C6-HSL, C8-HSL and 3-oxo-C4~C8 exogenous AHLs [19,20]. *A. tumefaciens* A136 bears the traI-lacZ fusion (pCF218) (pCF372) plasmids and capable of producing a blue colour from the hydrolysis of 5-bromo-4-chloro-3-indolyl-β-D-galactopyranoside by the β-galactosidase activity, in response to C8-HSL, 3-oxo-C8-HSL, C10-HSL, C12-HSL, 3-oxo-C12-HSL and C14-HSL exogenous AHLs molecule [7,20,21]. Stock cultures of the reporter strains were stored at −80 ºC in Luria-Bertani broth with 20% glycerol. The biosensor strains were aerobically activated in LB broth with shaking at 28 ºC for 24 h. When required, LB broth was supplemented with the appropriate amount of antibiotics to maintain the plasmids (*C. violaceum* CV026 with kanamycin 20 μg/mL; *A. tumefaciens* A136 with spectinomycin 50 μg/mL and tetracycline 4.5 μg/mL). The pH of the culture medium was adjusted to 7.0 ± 0.2 with 0.1 N solution of NaOH or HCL.

### 3.4. Collection of Activated Sludge

Activated sludge samples were collected in sterile umber color bottles from the MBR of GURI wastewater treatment plant, Guri-city, Gyeonggi-do, Republic of Korea in summer 2013. The collected sludge was transported to laboratory in ice box and extraction was carried out immediately. The pH of the MBR activated sludge samples was 6.74.

### 3.5. Isolation of Bacteria

Using a culture-dependent technique with various growth media, the isolation of bacteria from membrane bioreactor activated sludge was performed [4]. Different microbiological culture medium used in this study were LB broth (g L^−1^ of tryptone, 10.0; yeast extract, 5.0; sodium chloride, 10.0), NB (g L^−1^ of peptic digest of animal tissue, 5.0; sodium chloride, 5.0; beef extract, 1.5; yeast extract, 1.5), TSB (g L^−1^ of pancreatic digest of casein, 17.0; enzymatic digest of soybean meal, 3.0; dextrose, 2.5; sodium chloride, 5.0; dipotassium phosphate, 2.5), R2A broth (g L^−1^ of yeast extract, 0.5; protease peptone No.3, 0.5; casamino acids, 0.5; dextrose, 0.5; soluble starch, 0.5; sodium pyruvate, 0.3, dipotassium phosphate, 0.3; magnesium sulfate, 0.05) and synthetic wastewater (SW) (g L^−1^ of glucose, 1.0; yeast extrxct, 0.05; bactopeptone, 0.05; ammonium sulfate, 0.5; dipotassium hydrogen phosphate, 0.3; potassium dihydrogen phosphate, 0.3; magnesium sulphate heptahydrate, 0.009; iron (III) chloride hexahydrate, 0.0002; sodium chloride, 0.007; calcium chloride, 0.0002; sodium hydrogen carbonate 0.15). The activated sludge sample was diluted up to 10^−6^ with sterile distilled water and 100 μL was spread inoculated onto each growth medium agar plates. Such plates were then incubated at 28 ºC for 24 to 48 h and several morphologically distinct pure colonies were randomly chosen, subcultured on the same agar and grown in the same conditions. A total of 200 bacterial isolates, 40 from each medium agar plate, were selected and screened for AHLs production using two different biosensor systems. The stock cultures of bacterial isolates were maintained on LB agar at 4 ºC.

### 3.6. Screening of Bacterial Isolates for AHLs Production

As different biosensor systems detect specific types of AHLs, it is essential to use those biosensors that respond to a broad range of AHLs. Screening of all the bacterial isolates for production AHLs was carried out by agar plate diffusion assay with some modifications [19,22]. Briefly, active cultures of QS reporter bacterial strains were prepared in LB agar plates containing the antibiotics kanamycin 20 μg/mL for *C. violaceum* CV026 and spectinomycin 50 μg/mL and tetracycline 4.5 μg/mL for *A. tumefaciens* A136. The plates were incubated at 28 ºC for 24 h. A loopful of overnight grown cultures from both the plates were taken and separately added into 10 mL of sterile distilled water. Then 2.5 mL culture suspension of both biosensor strains were separately added to 100 mL of cooled LB medium (0.8% agar) kept at 45 ºC and poured as a thin plate. 40 μg/mL of X-gal as a visualizing agent was incorporated into LB medium used for *A. tumefaciens* A136. The bacterial isolates to be tested were grown in LB broth for 24 h and diluted to an OD 600 nm of 1.0 with sterile distilled water. About 5 μL of the test strains were then spot inoculated onto the surface of previously prepared chromoplates and allowed to dry. The petri plates were incubated for 24 to 48 h at 28 ºC and production of purple pigment violacein and blue coloration by β-galactosidase activity was observed as positive test for AHLs production.

### 3.7. Microtiter Plate Biofilm Formation Assay

AHLs producing bacterial isolates were evaluated for biofilm formation in 96-well flat bottom polystyrene microtiter plate (SPL Life Sciences, Pocheon-Si, Korea) by the method of O’Toole and Kolter [23] with slight modifications. The conditioning of microplate wells was carried out by inoculating 200 μL of LB broth for 1 h at room temperature. The wells were emptied and 190 μL of LB medium and 10 μL of bacterial cell suspensions (0.5 O.D._595nm_) were inoculated in each well. The plate was sealed with parafilm and incubated in static condition at 28 ºC for 24 and 48 h [24,25]. Following static incubation, the turbidity of the planktonic cultures was measured at 655 nm and the biofilm formation was quantified by classical crystal violet assay. Briefly, the supernatant from the microtiter plate wells was gently removed and the wells were rinsed thrice with 200 μL of sterile distilled water. The plate was dried in inverted position for 30 min and stained with 200 μL of 0.1% (*w*/*v*) crystal violet, per well, for 30 min. Excess crystal violet was removed from the wells and the biofilms were washed twice with distilled water. Finally, the absorbed crystal violet was extracted with 200 μL of 95% ethanol, per well for 1 h, and the O.D._595nm_ was measured with a plate reader (iMark™ Micrplate Absorbance Reader 168-1135, Bio-Rad, Hercules, CA, USA), which yields a measure of biofilm formation. A well with sterile LB broth served as controls.

### 3.8. Identification of AHLs Producing Bacteria

The screened AHLs producing bacterial isolates were identified through 16S rRNA gene sequences analysis. The bacterial genomic DNA was extracted using the GenElute™ Bacterial genomic DNA Kit (Sigma-Aldrich, St. Louis, MO, USA), according to the manufacturer’s instructions. 16S rRNA gene sequences were amplified with bacterial universal primers 518F (5′-CCAGCAGCCGCGGTAATACG-3′) and the reverse primer 800R (5′-TACCAGGGTATCTAATCC-3′). The purified polymerase chain reaction (PCR) amplicons were then sequenced and compared with the 16S rRNA gene sequences available in the GeneBank database at the National Centre for Biotechnology Information (NCBI) using the basic local alignment search tool BLASTN 2.2.28 algorithm [26].

### 3.9. Phylogenetic Analysis of AHLs Producing Bacteria Strains

All the 32 AHLs producing bacterial isolates nucleotide sequences were aligned with the program CLUSTAL W [27]. The aligned complete 16S rRNA sequences were subjected to phylogenetic analysis using the Molecular Evolutionary Genetic Analysis (MEGA) software version 4.0. The tree was generated with the Neighbor-Joining algorithm and bootstrap for 550 resamplings to ensure robustness and reliability of trees constructed [28].

### 3.10. Nucleotide Sequence Accession Number

The assembled complete 16S rRNA sequences of eleven distinguishable AHLs producing bacterial isolates have been deposited in public sequence repository NCBI GenBank using the BankIt sequence submission tool (http://www.ncbi.nlm.nih.gov/Genbank; National Institutes of Health, Rockville, MD, USA).

### 3.11. Extraction of AHLs from Activated Sludge

The crude extraction of AHLs from the MBR activated sludge from GURI wastewater treatment plant was carried out with acidified ethyl acetate [29]. Briefly, 20 mL of activated sludge was centrifuged at 9000 *g* for 10 min and the supernatant was mixed with an equal volume of acidified ethyl acetate (0.1% acetic acid). The mixture was then shaken at 180 rpm for 2 h at 25 ºC. After shaking, the upper organic layer was separated and freeze-dried under vacuum. The residue was then dissolved in 250 μL of acetonitrile and used further for chromatographic analysis.

### 3.12. Detection of Crude AHLs by Agar Plate Cross-Feeding Bioassay

In order to detect the occurrence of AHLs in activated sludge extract, the samples were analyzed by agar plate cross-feeding assay with two biosensor systems [21]. The LB agar plates containing kanamycin 20 μg/mL for *C. violaceum* CV026 and spectinomycin 50 μg/mL, tetracycline 4.5 μg/mL and X-gal 80 μg/mL for *A. tumefaciens* A136 were prepared. A loopful of crude AHLs extract was streak inoculated in parallel with approximately 1 cm to the *C. violaceum* CV026 and *A. tumefaciens* A136 biosensor strains on respective agar plates. After incubation for overnight at 28 ºC the plates were observed for activation of LuXR homologue CviR and traI-lacZ fusion in *C. violaceum* CV026 and *A. tumefaciens* A136 reporter strains respectively to confirm the presence of AHLs. The occurrence of AHLs in crude extract is indicated by the development of purple and blue coloration of *C. violaceum* CV026 and *A. tumefaciens* A136 streaked lines respectively.

### 3.13. Thin Layer Chromatography (TLC) for AHL Identification

Ten microlitre of crude sludge AHLs preparation was spotted on a C18 reverse-phase TLC plate (60RP-18F_254_S, Merck, Germany) and the chromatogram was developed in methanol: water (60:40) solvent system until the solvent front line reaches up to 1 cm from top edge [7,29]. After being completely dried in clean bench for 20 min, the TLC plate was separately overlaid with 1.5% LB agar containing *C. violaceum* CV026 (kanamycin 20 μg/mL) and *A. tumefaciens* A136 (spectinomycin 50 μg/mL, tetracycline 4.5 μg/mL and X-gal 80 μg/mL). The commercially available AHLs prepared in acetonitrile were used as reference standards. TLC plate loaded with crude AHLs extract and biosensor strains was incubated overnight at 28 ºC and observed for the apparent of purple and blue spots respectively. The results were captured digitally and the Rf value of standard AHLs, defined by the ratio of the distance traveled by spot and that of solvent front were calculated. The approximate type of extracted sludge AHLs were determined by comparing the Rf values with those of standard reference AHLs.

### 3.14. High Performance Liquid Chromatography (HPLC) Analysis of AHLs

Analysis of AHLs extract was performed on an Agilent 1200 HPLC system (Agilent, Santa Clara, CA, USA) equipped with a ZORBAX Eclipse XDB-C18 column (4.6 mm × 150 mm, 5 μm particle size) kept at 30 ºC. Commercially available standard AHLs viz. C4-HSL, C6-HSL, C8-HSL, 3-oxo-C8-HSL, C10-HSL, C12-HSL, 3-oxo-C12-HSL and C14-HSL were dissolved in acetonitrile to make 1000 μg/mL of stock solutions. Each of the eight AHLs were further diluted in acetonitrile to obtain 10, 20, 30, 40 and 50 μg/mL of individual and its mixtures working solutions. The resulting AHLs standards, its mixture, crude sludge extract and acetonitrile as blank were injected into a column at a flow rate of 0.25 mL/min. The HPLC conditions and instrument parameters as previously described includes an isocratic profile of methanol/water (35:65, *v/v*) for five min, followed by a linear gradient from 35% to 95% methanol in water over 33 min. A subsequent linear gradient from 95% to 35% methanol in water over 2 min, and an isocratic profile of methanol/water (35:65, *v/v*) for five min were applied for flushing the column for the following run [21,30].

### 3.15. Statistical Analysis

Statistical analysis of biofilm formation was performed using SPSS software (IBM Corporation, Armonk, NY, USA). The values presented are means of four repeated experiments with standard deviations indicated in error bars.

## 4. Conclusions

This study represents the culture-dependent molecular approach to elucidate the molecular diversity and phylogenetic relationship of AHLs producing bacterial communities in a MBR treating wastewater. Our data have shown that AHLs producing bacteria were distinct from each other and reflect the presence of various genera. 16S rRNA gene sequence characterization indicates the predominance and thus the potential role of *Aeromonas* and *Enterobacter* species in AHLs production and subsequent biofilm formation. Furthermore, evidence for the presence of broad range of AHLs in activated sludge extract and the ability of respective bacterial isolates to produce such AHLs suggest that these genera could be main targets for fouling control in MBR. This study is an important step towards exploring the future QSI strategies to control biofouling in MBR treating wastewater.

## Figures and Tables

**Table 1.** Screening of bacterial isolates for AHLs production using violacein and β-galactosidase assay with *C. violaceum* CV026 and *A. tumefaciens* A136 reporter strains.

Isolate numbers	Isolate identifier	Reporter strains	

		*C. violaceum* CV026	*A. tumefaciens* A136
1	NA1	+++	+++
2	NA2	++	+++
3	NA3	--	++
4	NA4	++	+++
5	NA5	--	++
6	NA6	--	++
7	LBA1	+++	+++
8	LBA2	++	+++
9	LBA3	--	+++
10	LBA4	--	+++
11	LBA5	--	++
12	TSA1	+++	+++
13	TSA2	+++	+++
14	TSA3	--	+++
15	TSA4	--	++
16	TSA5	--	++
17	TSA6	--	++
18	TSA7	--	++
19	TSA8	--	+++
20	R2A1	+++	+++
21	R2A2	+++	+++
22	R2A3	+++	++
23	R2A4	--	++
24	R2A5	--	++
25	R2A6	+++	+++
26	SWA1	++	+++
27	SWA2	--	+++
28	SWA3	--	++
29	SWA4	--	+++
30	SWA5	--	+++
31	SWA6	++	++
32	SWA7	--	++

++ Medium color production;

+++ Strong color production.

**Table 2.** Nucleotide blast analysis of 16S rRNA gene sequences from AHLs producing bacterial isolates using BLASTN Algorithm.

Isolate identifier	Sequence length (bp)	Max score	Identity (%)	Closest relative strain at database and its description	Accession number
NA1	1469	2678	99	*Aeromonas hydrophila* subsp. *hydrophila* ATCC 7966 strain ATCC 7966 16S rRNA, complete sequence	NR_074841.1
NA2	1470	2715	100	*Aeromonas media* strain RM 16S rRNA, partial sequence	NR_036911.2
NA3	1465	2684	100	*Aeromonas hydrophila* subsp. *hydrophila* ATCC 7966 strain ATCC 7966 16S rRNA, complete sequence	NR_074841.1
NA4	1464	2575	99	*Enterobacter cancerogenus* strain LMG 2693 16S rRNA, partial sequence	NR_044977.1
NA5	1474	2712	100	*Aeromonas hydrophila* subsp. *hydrophila* ATCC 7966 strain ATCC 7966 16S rRNA, complete sequence	NR_074841.1
NA6	1465	2684	100	*Aeromonas media* strain RM 16S rRNA, partial sequence	NR_036911.2
LBA1	1461	2641	100	*Aeromonas hydrophila* subsp. *anaerogenes* strain CECT 4221 16S rRNA, partial sequence	NR_104824.1
LBA2	1474	2684	99	*Aeromonas hydrophila* subsp. *dhakensis* strain P21 16S rRNA, partial sequence	NR_042155.1
LBA3	1396	2518	100	*Enterobacter sp.* 638 strain 638 16S rRNA, complete sequence	NR_074777.1
LBA4	1383	2505	100	*Enterobacter cancerogenus* strain LMG 2693 16S rRNA, partial sequence	NR_044977.1
LBA5	1459	2586	99	*Enterobacter ludwigii* strain EN-119 16S rRNA, complete sequence	NR_042349.1
TSA1	1471	2717	100	*Aeromonas hydrophila* subsp. *anaerogenes* strain CECT 4221 16S rRNA, partial sequence	NR_104824.1
TSA2	1476	2726	100	*Aeromonas hydrophila* subsp. *anaerogenes* strain CECT 4221 16S rRNA, partial sequence	NR_104824.1
TSA3	1460	2601	100	*Pseudomonas japonica* strain IAM 15071 16S rRNA, partial sequence	NR_040992.1
TSA4	1459	2623	100	*Leclercia adecarboxylata* strain CIP 82.92 16S rRNA, complete sequence	NR_104933.1
TSA5	1462	2647	99	*Enterobacter cancerogenus* strain LMG 2693 16S rRNA, partial sequence	NR_044977.1
TSA6	1462	2558	100	*Leclercia adecarboxylata* strain CIP 82.92 16S rRNA, complete sequence	NR_104933.1
TSA7	1461	2671	100	*Raoultella ornithinolytica* B6 strain B6 16S rRNA, complete sequence	NR_102983.1
TSA8	1463	2680	100	*Serratia marcescens* WW4 strain WW4 16S rRNA, complete sequence	NR_102509.1
R2A1	1475	2719	100	*Aeromonas hydrophila* subsp. *anaerogenes* strain CECT 4221 16S rRNA, partial sequence	NR_104824.1
R2A2	1465	2706	100	*Aeromonas hydrophila* subsp. *anaerogenes* strain CECT 4221 16S rRNA, partial sequence	NR_104824.1
R2A3	1476	2699	99	*Aeromonas hydrophila* subsp. *hydrophila* ATCC 7966 strain ATCC 7966 16S rRNA, complete sequence	NR_074841.1
R2A4	1455	2599	99	*Enterobacter ludwigii* strain EN-119 16S rRNA, complete sequence	NR_042349.1
R2A5	1465	2686	99	*Citrobacter freundii* strain DSM 30039 16S rRNA, partial sequence	NR_028894.1
R2A6	1464	2638	99	*Enterobacter ludwigii* strain EN-119 16S rRNA, complete sequence	NR_042349.1
SWA1	1490	2571	98	*Enterobacter ludwigii* strain EN-119 16S rRNA, complete sequence	NR_042349.1
SWA2	1460	2676	99	*Klebsiella variicola* At-22 strain At-22 16S rRNA, complete sequence	NR_074729.1
SWA3	1450	2555	100	*Enterobacter ludwigii* strain EN-119 16S rRNA, complete sequence	NR_042349.1
SWA4	1462	2619	99	*Leclercia adecarboxylata* strain CIP 82.92 16S rRNA, complete sequence	NR_104933.1
SWA5	1458	2619	99	*Enterobacter ludwigii* strain EN-119 16S rRNA, complete sequence	NR_042349.1
SWA6	1462	2673	100	*Serratia marcescens* WW4 strain WW4 16S rRNA, complete sequence	NR_102509.1
SWA7	1463	2673	99	*Serratia marcescens* WW4 strain WW4 16S rRNA, complete sequence	NR_102509.1
